# Patient and Provider Perspectives on the Patient Journey in Immunoglobulin A Nephropathy

**DOI:** 10.1016/j.xkme.2026.101242

**Published:** 2026-01-06

**Authors:** Abdallah S. Geara, Kelly B. Chen, Whitney Simmons

**Affiliations:** 1 University of Pennsylvania Perelman School of Medicine, Philadelphia, PA; 2 Stanford Health Care, Stanford, CA; 3 Mosaic Infusion Solutions, Amerita Inc., Oklahoma City, OK

**Keywords:** advocacy, clinical journey, IgA nephropathy, management, patient, patient journey, patient perspective, patient self-advocacy, provider, provider perspective, shared decision making

## Abstract

The clinical presentation and disease course of immunoglobulin A nephropathy (IgAN), the most common primary glomerular disease worldwide, vary considerably from patient to patient, often prolonging and complicating the diagnostic process. Additionally, the IgAN treatment landscape is rapidly changing, with ongoing development of numerous new agents targeting the underlying disease mechanism for greater efficacy. The variability of IgAN disease presentation and progression, the often complex path to diagnosis, and the evolving treatment landscape may pose significant challenges for patients and health care providers along the clinical journey. Furthermore, many factors associated with the health care system, providers, and patients may affect IgAN diagnosis, treatment, and management. This report uses the clinical and personal experiences and perspectives of the authors—a nephrologist treating patients with IgAN, an ambulatory nephrology nurse practitioner diagnosed with IgAN, and an ambulatory infusion center nurse practitioner diagnosed with IgAN—to depict the patient journey with this condition from the first clinical presentation through to long-term management. The clinical journey may be improved by increasing provider awareness of IgAN and its patient impact, as well as encouraging self-advocacy among patients.

## Introduction

Immunoglobulin A nephropathy (IgAN) is a progressive autoimmune disease affecting the kidneys. In IgAN, immune complexes composed of galactose-deficient immunoglobulin A1 and its autoantibodies accumulate in the glomeruli.^1^^,^^2^ Immune complex accumulation triggers intermittent or chronic glomerular inflammation,^1^^,^^3^^,^^4^ resulting in glomerular and tubulointerstitial scarring over time.^3^ Without effective treatment, IgAN can progress to kidney failure within the lifetime of many patients.^5^

In the United States (US), approximately 13 cases of IgAN per million people are reported annually, with most patients diagnosed by 40 years of age.^6^, ^7^, ^8^ However, IgAN prevalence estimates vary worldwide because of genetic diversity, different screening and diagnostic protocols, and socioeconomic factors affecting health care access.^8^, ^9^, ^10^ Given the often asymptomatic presentation and the requirement of a kidney biopsy for diagnosis, IgAN prevalence is likely underestimated globally.^9^

The clinical presentation and disease course of IgAN vary considerably among patients.^11^ While some individuals present early with signs like hematuria and/or proteinuria found incidentally on routine health screenings, others present in late stages with advanced chronic kidney disease.^11^ This lack of straightforward signs may contribute to a prolonged, convoluted path to IgAN diagnosis depending on provider familiarity with the disease.^12^^,^^13^ Once the diagnosis is confirmed, each patient must receive appropriate treatment. Historically, IgAN treatment focused on providing supportive care, including controlling blood pressure to reduce proteinuria,^14^ but recent research advancements have enabled the development of agents targeting the underlying disease mechanisms for greater efficacy.^3^

The variability of IgAN presentation and disease progression, the often complex clinical journey to diagnosis, and the rapidly changing treatment landscape create challenges for patients and health care providers.^3^^,^^12^^,^^13^ This report uses the authors’ clinical and personal experiences to describe the current, often convoluted patient journey in IgAN, with the hope of improving it by increasing disease awareness and highlighting its patient impact. Dr. Abdallah S. Geara, MD, is a nephrologist and the Clinical Director of the Glomerular Disease Program at the University of Pennsylvania Hospital in Philadelphia, PA. Kelly B. Chen, MSN, AGACNP-BC, is an ambulatory nephrology nurse practitioner at Stanford Health Care in Stanford, CA, diagnosed with IgAN in May 2018. Whitney Simmons, MSN, APRN-CNP, IgCN, CRNI, is a nurse practitioner at Mosaic Infusion Solutions in Oklahoma City, OK, diagnosed with IgAN in July 2012. To develop this report, the authors collaboratively identified key themes of importance to patients and health care providers within the IgAN clinical journey; each author was then individually interviewed to obtain their unique perspectives on these topics. Direct quotes from the authors that strongly conveyed or supported the concepts being presented were used within the report text.

## The Patient Journey in IgAN

**Figure 1:**
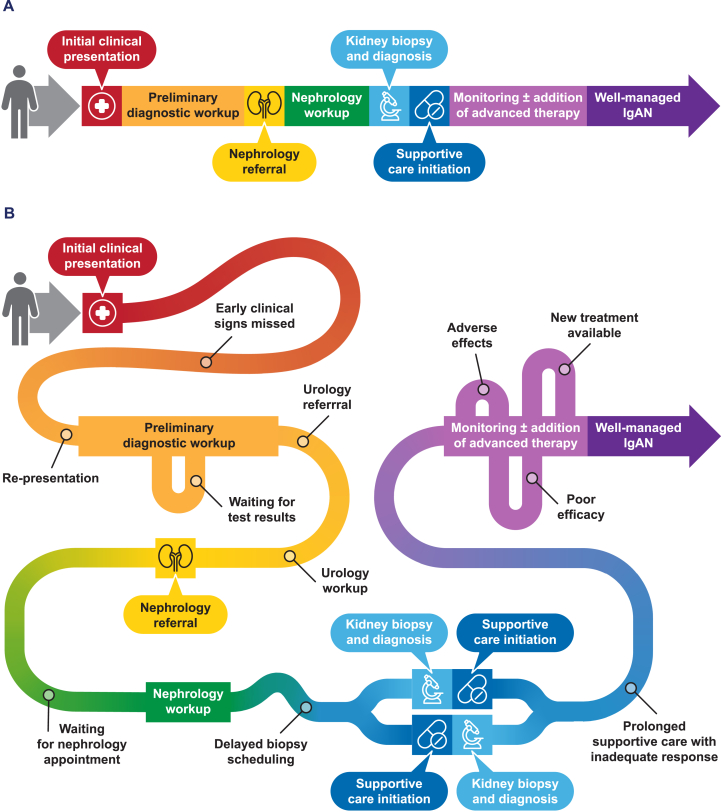
The patient journey in immunoglobulin A nephropathy (IgAN). (A) Ideal patient journey. (B) Realistic patient journey.

Figure 1A and B depicts hypothetical ideal and realistic patient journeys in IgAN and was developed based on the clinical and personal experiences of the authors. Though the journey varies for each patient,^11^, ^12^, ^13^ its key steps include initial presentation, preliminary diagnostic workup, nephrology referral, kidney biopsy, supportive care initiation, potential addition of targeted therapeutics, and long-term monitoring.

### Diagnosis of IgAN

#### Initial Clinical Presentation and Preliminary Diagnostic Workup

Many patients with IgAN are asymptomatic before diagnosis,^14^ with signs of disease found incidentally on routine health screenings often performed by a primary care provider (PCP).^11^^,^^15^^,^^16^ When IgAN symptoms are present, they vary; patients may notice visible hematuria (possibly following a respiratory or gastrointestinal infection), foamy urine, fatigue, edema, or hypertension.^11^^,^^16^

After initial presentation, the preliminary diagnostic workup for IgAN also varies.^12^^,^^16^ Ideally, patients presenting with hematuria and/or proteinuria on urinalysis should undergo thorough medical history review, physical examination, and urine microscopy to confirm the findings and rule out serious underlying causes, such as urinary tract cancer.^17^^,^^18^ Other preliminary diagnostics may include bloodwork, blood pressure evaluation, urine culture, and/or proteinuria quantification as appropriate.^17^^,^^18^

Need for urology and/or nephrology referral should be based on these initial assessments. Persistent proteinuria, urinary red blood cell abnormalities, cellular casts, or reduced kidney function are some indications for nephrology referral.^17^^,^^18^ However, affected patients may initially be referred to urology,^16^ delaying IgAN diagnosis. For example, when Whitney Simmons presented with sustained proteinuria on urinalysis, her PCP first referred her to a urologist, and she underwent a cystoscopy and proteinuria quantification before nephrology referral.

#### Nephrology Referral and Kidney Biopsy

During nephrology evaluation, patients may undergo repeat urine and blood testing, proteinuria quantification, evaluation of glomerular filtration rate, genetic and/or immunologic testing, kidney imaging, and/or kidney biopsy.^19^ A kidney biopsy is required for definitive IgAN diagnosis^20^ and is typically performed by a nephrologist or interventional radiologist.^12^^,^^21^ However, this procedure may be delayed because of the lack of national or global consensus regarding biopsy indications, contraindications (eg, bleeding risk, poorly controlled hypertension, or infection), risk of complications, comorbid conditions (eg, obesity), poor accessibility of specialty care, or absence of severe clinical signs.^22^, ^23^, ^24^, ^25^

Delays occur frequently during the journey to IgAN diagnosis, making this process prolonged and complicated (Fig 1B).^12^^,^^13^ In a population of US adults with IgAN, the median time from first clinical sign to diagnosis was 5.0 months.^13^ Waiting to conduct tests, waiting for specialist referral, waiting for test results, and patients’ inability to consult sooner are common reasons for diagnostic delay.^12^

I was in nurse practitioner school, so life was busy, and I had to delay the [IgAN] workup a lot. The 24-hour urine protein measurement was especially time-consuming and takes a full day to do, and then we repeated it again to confirm. It wasn’t until after all those results came back [that] I got my referral. The other biggest delay was [that] I had to wait 2–3 months for my initial nephrology appointment. – Kelly Chen

Because delays in IgAN diagnosis may worsen patient outcomes,^5^^,^^13^ timely and appropriate evaluation of at-risk patients is vital.

### IgAN Treatment and Monitoring

#### Supportive Care to Manage Generic Responses to IgAN-Induced Nephron Loss

The treatment of IgAN may employ multiple medications to address all disease components.^26^ Per the KDIGO (Kidney Disease: Improving Global Outcomes) Clinical Practice Guideline, initial IgAN treatment involves the implementation of supportive care to manage the consequences of existing IgAN-induced nephron loss by controlling blood pressure, reducing proteinuria, and preserving kidney function.^20^ Traditionally, supportive care in IgAN has encompassed renin–angiotensin–aldosterone system inhibition, lifestyle changes (ie, weight loss, exercise, low-sodium diet, and smoking cessation), and management of any cardiovascular risk.^14^ In recent years, novel agents, including sodium-glucose cotransporter-2 inhibitors, dual endothelin and angiotensin II receptor antagonists, and endothelin A receptor antagonists, have also been found effective in IgAN by reducing glomerular hyperfiltration and proteinuria.^20^^,^^27^, ^28^, ^29^, ^30^ Two such agents (sparsentan [dual endothelin and angiotensin II receptor antagonist] and atrasentan [endothelin A receptor antagonist]) were recently approved by the US Food and Drug Administration for the treatment of adults with IgAN.^31^^,^^32^ Optimization of supportive care through the utilization of these various agents remains a core pillar of IgAN treatment.^20^

#### Therapies Addressing IgAN-Specific Drivers of Nephron Loss

In addition to supportive care, which manages the consequences of existing IgAN-induced nephron loss, patients at risk of disease progression should also receive treatments to address ongoing IgAN-specific drivers of nephron loss. Traditionally, immunosuppressive agents, such as corticosteroids, have demonstrated success for this purpose.^33^, ^34^, ^35^ However, corticosteroids do not specifically target the underlying disease mechanism, may have inconsistent efficacy in this patient population, and can cause serious side effects like infection or impaired glucose metabolism.^3^^,^^14^^,^^33^^,^^36^

Although the IgAN disease mechanism is still being studied, it is thought to involve 4 steps: (1) production of galactose-deficient immunoglobulin A1 and its entry into the circulation, (2) autoantibody production and recognition of the galactose-deficient immunoglobulin A1, (3) formation of circulating immune complexes, and (4) immune complex deposition in the glomeruli, which activates pathways leading to glomerular inflammation and progressive kidney damage.^1^^,^^2^

In recent years, multiple new drugs targeting this disease mechanism have been investigated in an effort to achieve more effective and safer IgAN treatment.^3^ Currently, 3 such agents—iptacopan (inhibitor of factor B of the alternative complement pathway), a targeted-release formulation of budesonide (corticosteroid), and sibeprenlimab (A Proliferation-Inducing Ligand Blocker)—are US Food and Drug Administration–approved for use in IgAN.^37^, ^38^, ^39^ Many other targeted agents remain in development.^3^ Targeted-release budesonide, which was US Food and Drug Administration–approved before the 2025 KDIGO Guideline cutoff date of July 2024, was included in the updated treatment algorithm for IgAN and is currently recommended as the first-line treatment for patients at risk of progressive loss of kidney function.^20^ Clinical trial enrollment may also be considered for all patients, especially if immunomodulatory therapy with the available agents is unsuccessful, contraindicated, or undesirable.^3^^,^^20^

While offering hope for more effective therapies, the changing IgAN treatment landscape may also create challenges for providers and patients.^3^^,^^40^ The novelty of these drugs, the absence of guidelines defining where all agents fit in the treatment approach, and the uncertainty regarding which patients may respond best to which agents may complicate treatment decisions.^3^^,^^26^

As a physician, the availability of novel therapy and the continuously changing standard of care is creating a challenge in identifying appropriate patients for referral to clinical trials. – Dr. Abdallah Geara

From a patient perspective, we also struggle. Do I move [toward] a cocktail of marketed therapies, randomize myself into a new trial, or a combination of both? Nobody knows which one is better for whom. – Kelly Chen

Potential side effects, the need to stop current treatments, and evidence of drug efficacy for disease management in previous trials are major considerations affecting patients’ willingness to enroll in clinical trials.^16^ In the authors’ experiences, even when patients elect to enroll, the screening process may be prolonged (weeks to months), during which IgAN continues to progress, and patients may be excluded based on their current therapies or clinical characteristics.

The uncertainty surrounding the recommended treatment approach may also result in frequent treatment switching as providers attempt to manage IgAN while mitigating adverse effects. For example, Whitney Simmons has received various on- and off-label IgAN treatments since her diagnosis, many of which had limited success or caused side effects. Since her disease became poorly controlled after the birth of her second child in 2020, at which time she was taking a pregnancy regimen including a calcineurin inhibitor, beta-blocker, diuretic, and fish oil supplement, she has tried (in no specific order) an angiotensin-converting enzyme inhibitor, systemic and locally acting corticosteroids, a different calcineurin inhibitor, antimetabolites, a dual endothelin and angiotensin II receptor antagonist, a sodium-glucose cotransporter-2 inhibitor, an antimalarial drug, a statin, and vitamin D3 supplementation. Amid these treatment trials, she also underwent a second kidney biopsy and switched nephrologists to attempt to achieve good disease control.

Providers and patients must actively self-educate in this evolving IgAN treatment landscape to stay current. In a global survey of nephrologists, only 30% of respondents reported enrolling patients in clinical trials, and up to 60% reported a lack of awareness of novel drug clinical trials conducted in their countries.^35^ However, provider knowledge is essential because patients often rely on them for disease education and treatment guidance.^41^, ^42^, ^43^ From the patient's perspective, the lack of understandable resources about IgAN and its treatments may prevent patients from making fully informed care decisions.^15^^,^^44^ In a focus group of patients with IgAN, 48% of respondents had not participated in clinical trials because they were unaware of the opportunity.^16^

## Challenges in IgAN Management

**Figure 2:**
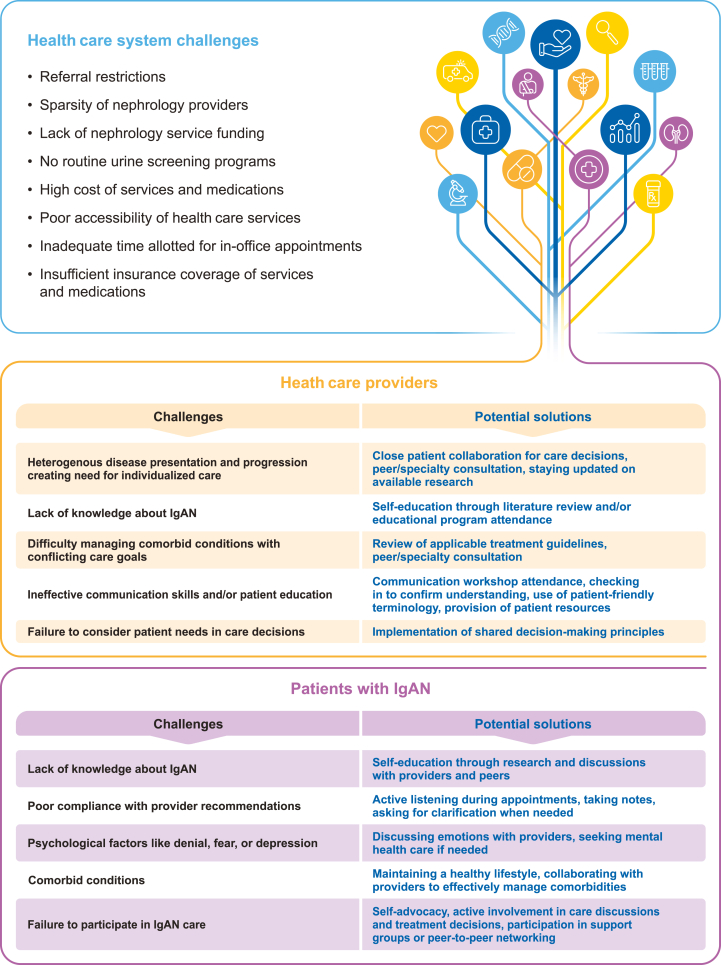
Challenges in immunoglobulin A nephropathy (IgAN) management and potential solutions for health care providers and patients.

In addition to the changing treatment landscape, various challenges associated with the health care system, health care providers, and patients may also affect the clinical journey in IgAN (Fig 2).

### Health Care System Challenges

Patients and providers must operate within the limits of their health care systems, which often impose restrictions affecting IgAN management.^45^ Health care system limitations affecting IgAN diagnosis and treatment may include a lack of urine screening programs, poor accessibility of health care services, high cost and/or insufficient insurance coverage of diagnostics and treatments, referral restrictions, scarcity of nephrology providers, and inadequate time allotted for in-office consultations.^8^^,^^35^^,^^40^^,^^45^, ^46^, ^47^

In the US, there are an estimated 2,000+ patients with chronic kidney disease per nephrology provider,^40^ which limits the capacity of nephrologists to care for patients with IgAN or other kidney diseases, especially in early disease.^40^^,^^48^

Our nephrology clinics are always booked. Almost all nephrology [clinics] are booked 3 to 6 months ahead, and that creates risk for patients not to follow [up]. – Dr. Abdallah Geara

This shortage of nephrology providers, which is escalating over time,^48^ may prevent or complicate referral, delay appointment scheduling, and constrain the duration of in-office appointments.^40^^,^^45^ In the authors’ experiences, patient/caregiver education and IgAN management decisions mainly occur during in-office appointments, so sufficient time must be allotted for these conversations. A nephrologist shortage also places the burden of kidney disease management on PCPs, who are generally less familiar with these disorders and often have large caseloads.^40^^,^^45^ In a survey of US PCPs, 38% of respondents saw over 100 patients per week.^40^

Establishing routine urine screening programs may also facilitate early IgAN detection and treatment.^49^ IgAN is diagnosed earlier and at higher rates in countries that perform annual urine screening (eg, Japan, Taiwan, and South Korea),^8^^,^^9^^,^^49^ but these programs have not been implemented in most Western countries.^9^

### Health Care Provider Challenges

Health care provider factors that may affect the patient journey in IgAN include poor knowledge about IgAN, difficulty managing multiple comorbid conditions, ineffective communication skills and/or patient education, poor consideration of patient needs in care decisions, and the historic view of IgAN as a relatively benign disease.^40^^,^^41^^,^^44^^,^^45^^,^^50^

Because IgAN is rare and often asymptomatic,^8^^,^^14^ referring physicians may miss early signs and fail to recommend additional workup.^45^^,^^51^ Those who notice the signs may delay diagnosis with non-indicated tests or referrals,^45^^,^^52^^,^^53^ or may be unsure whether referral is indicated and attempt to manage patients themselves.^45^

I saw my PCP three times before I got a referral. Could I have gotten a referral after that first [urinalysis]? It’s just something I think about. – Kelly Chen

I think PCPs are not familiar with IgAN, and I do not expect them to be familiar with it. I do expect them to be concerned by the presence of hematuria or proteinuria at any level, so they can send these patients for further urology or nephrology evaluation. – Dr. Abdallah Geara

This highlights the importance of kidney disease education for PCPs and other referring providers, to encourage early recognition, referral, and treatment of patients with IgAN and other kidney diseases.^40^^,^^45^^,^^54^

For optimal IgAN management, providers must also consider that their treatment goals, such as improving clinical laboratory parameters, may not match those of their patients. Providers should try to prioritize outcomes valued by patients, like symptom control, improved long-term disease outcome, greater life participation, and ability to plan for a family.^16^^,^^44^^,^^55^

How patients feel and being able to treat with future goals in mind is very important. What is [most] important to [patients] is not always what is most important to providers. – Whitney Simmons

I think there’s a disconnect. The nephrologists are worried about our kidneys, but we’re worried about, “Can I have a normal life?” – Kelly Chen

Holistic, patient-centered care can produce higher levels of patient compliance, improved patient self-management, better patient recall of important treatment information, improved patient mental and physical health, and greater patient empowerment.^43^

Maintaining a strong patient–provider partnership during IgAN diagnosis and management, through effective communication and implementation of shared decision-making principles, is also important to improve the clinical journey and promote positive outcomes.^25^^,^^56^

The best thing I ever did was switch to my current [physician]. He allows me to provide input, make suggestions, and even allows my ideas to be incorporated. He allows me to express my concerns, and he addresses them. He will research anything that I recommend and come back with a scientific-based answer, and not just, “Well, I’ve never done that,” or “I’m not sure about it.” – Whitney Simmons

### Patient Challenges

Patient factors that may affect the clinical journey in IgAN include inadequate disease knowledge; poor compliance with provider recommendations; psychological factors such as denial, fear, or depression; comorbid conditions; and failure to participate in care.^16^^,^^25^^,^^40^^,^^42^^,^^43^^,^^45^

Lack of understanding about their disease, especially in those without noticeable symptoms, may cause patients to postpone care until IgAN is more advanced.^15^^,^^25^^,^^40^ Patients may also delay for psychological or emotional reasons, or if they believe that progression to kidney failure is inevitable.^42^

Per social norms, patients traditionally assume passive roles with their health care providers.^43^ In a study of clinical interactions between patients with chronic kidney disease and their PCPs, only 15% of patients actively participated in discussions about chronic kidney disease with their provider.^41^ However, patient self-advocacy and participation in disease management can help improve patient–provider relationships and optimize health outcomes.^43^

### THE PATIENT IMPACT OF IgAN

Living with IgAN imparts significant burdens on many aspects of patients’ lives.^8^^,^^16^^,^^57^ Physical burdens include disease symptoms and/or medication side effects, like fatigue, edema, pain, high blood pressure, gastrointestinal issues, sleep disturbances, weight gain, and temperature intolerance.^15^^,^^16^^,^^55^^,^^57^, ^58^, ^59^, ^60^, ^61^, ^62^ Mental and emotional burdens associated with IgAN include stress, anxiety, depression, fear, sadness, helplessness, guilt, mood fluctuations, brain fog, and low self-esteem.^15^^,^^16^^,^^55^^,^^57^^,^^60^^,^^62^^,^^63^ IgAN may affect social health by causing feelings of loneliness, limiting participation in social activities, and creating relationship strain.^15^^,^^16^^,^^55^

[IgAN] prevents you from being as social as you want to be. Usually, social settings [involve] high salt, high carb meals, perhaps there’s alcohol…We’re not chatting and catching up with friends or family while eating fruits and vegetables. – Kelly Chen

Patients may also be financially affected because of inability to work, impaired work performance, loss of employment, inability to afford medical insurance, or IgAN health care costs.^16^^,^^57^^,^^58^^,^^60^^,^^64^^,^^65^ Lifestyle changes, such as dietary and activity restrictions, may be necessary for effective IgAN management but reduce patient quality of life.^55^^,^^60^^,^^66^

Another important aspect of IgAN is its potential to hinder patients’ future plans or goals.^55^

When I was diagnosed, I felt like I might not be able to achieve all the goals I wanted to achieve in my life, that IgAN would be a burden on me forever. It took a few years for me to accept this reality. – Kelly Chen

IgAN and some of its treatments, including mycophenolate mofetil, cyclophosphamide, sparsentan, atrasentan, empagliflozin, and dapagliflozin, may impair fertility and/or increase maternal and fetal risks of pregnancy,^31^^,^^32^^,^^55^^,^^67^, ^68^, ^70^, ^69^, ^71^ which can affect family planning and cause emotional hardship for patients and their loved ones.

[My prior physician] told me that it’s likely that I won’t be able to have children. I was engaged and 23 at the time, so this was very difficult. – Whitney Simmons

In addition, the need for continuous care with trusted providers may restrict patients’ abilities to travel or relocate.

My now-husband moved to Shanghai in 2020 for work, so I planned to join him there, but had to come back to the United States to continue treatment. I ended up feeling really disappointed in myself that my disease was taking that freedom away from me. – Kelly Chen

Because all these burdens contribute to the patient experience of IgAN and can affect the clinical journey, providers must consider them for effective disease management.

## Conclusion

For many patients with IgAN, establishment of a definitive diagnosis may be delayed because of asymptomatic presentation, ambiguous clinical signs, and the need for kidney biopsy. However, timely identification and nephrology referral of at-risk patients facilitates prompt diagnosis and treatment initiation to reduce the patient burden of IgAN and optimize outcomes. As the IgAN treatment landscape changes, it is important that providers stay current on available therapies and clinical trials, and that patients and providers collaborate to promote patient engagement in care. Provider and patient awareness of the various challenges in the IgAN clinical journey will help facilitate effective navigation for improved disease management.

As evidenced by the perspectives in this report, the clinical journey in IgAN varies for each patient. A limitation of this study is that both patient authors are also US health care providers; thus, their experiences may differ considerably from those of patients less familiar with the US health care system. Additionally, while many of the challenges described in this report may be common to all patients, diversity of experiences is expected based on geographic, socioeconomic, and health care system factors. Despite this limitation, amplifying the patient voice in IgAN, as achieved here, can only help improve disease awareness, empower others through education, and promote the optimization of care for the IgAN population.
